# *Staphylococcus lugdunensis* Endocarditis in a 35-Year-Old Woman in Her 24th Week of Pregnancy

**DOI:** 10.1155/2016/7030382

**Published:** 2016-03-09

**Authors:** Mounir Khafaga, Karl-Patrik Kresoja, Berndt Urlesberger, Igor Knez, Philipp Klaritsch, David Benjamin Lumenta, Robert Krause, Dirk von Lewinski

**Affiliations:** ^1^Department of Cardiology, Medical University of Graz, 8036 Graz, Austria; ^2^Division of Neonatology, Department of Paediatrics, Medical University of Graz, 8036 Graz, Austria; ^3^Division of Cardiac Surgery, Department of Surgery, Medical University of Graz, 8036 Graz, Austria; ^4^Department of Obstetrics and Gynecology, Medical University of Graz, 8036 Graz, Austria; ^5^Division of Plastic, Aesthetic and Reconstructive Surgery, Department of Surgery, Medical University of Graz, 8036 Graz, Austria; ^6^Section of Infectious Diseases and Tropical Medicine, Department of Internal Medicine, Medical University of Graz, 8036 Graz, Austria

## Abstract

*Background*. Infective endocarditis is associated with considerable morbidity and mortality. Guidelines addressing prophylaxis and management of infective endocarditis do not extensively deal with concomitant pregnancy, and case reports on infective endocarditis are scarce. This is the first published report of infective endocarditis by* Staphylococcus lugdunensis* in a pregnant woman.* Case Presentation*. We report a single case of a 35-year-old woman in her 24th week of pregnancy who was admitted to our intensive care unit with fever and suspected infectious endocarditis. Blood culture detected* Staphylococcus lugdunensis*. A vegetation and severe mitral regurgitation due to complete destruction of the valve confirmed the diagnosis. An interdisciplinary panel of cardiologists, maternal-fetal medicine specialists, cardiac and plastic surgeons, infectiologists, anesthesiologists, and neonatologists was formed to determine the best therapeutic strategy.* Conclusions*. Timing and indications for surgical intervention to prevent embolic complications in infective endocarditis remain controversial. This original case report illustrates how managing infective endocarditis by* Staphylococcus lugdunensis* particularly in the 24th week of pregnancy can represent a therapeutic challenge to a broad section of specialties across medicine. Critical cases like this require a thorough weighing of risks and benefits followed by swift action to protect the mother and her unborn child.

## 1. Introduction

Despite the advances in medical, surgical, and critical care, infective endocarditis remains a disease that is associated with considerable morbidity and mortality [1]. Early and appropriate antimicrobial treatment is critical to avoid neurological complications in infective endocarditis [2]. Many factors affect the outcome of this serious disease, including virulence of the microorganism, characteristics of the patient, presence of underlying disease, delays in diagnosis and treatment, surgical indications, and timing of surgery [1]. Though professional societies have published guidelines addressing prophylaxis and management of infective endocarditis [3–7], they do not decisively deal with concomitant pregnancy and relevant case reports are scarce. Moreover, surgical controversies regarding indication and timing of treatment exist, especially in pregnancy [8]. We describe the case of a 35-year-old woman in her 24th week of pregnancy who was admitted to our intensive care unit with fever and suspected infective endocarditis.

## 2. Case Presentation

A 35-year-old pregnant woman with fever and suspected infective endocarditis was referred to our intensive care unit from a peripheral hospital after she had undergone a wedge excision for paronychia of the right great toe three days earlier. It was her fifth pregnancy, preceded by two terminations of pregnancy and two spontaneous births of two healthy children. Apart from a bilateral breast augmentation with implants no other relevant past medical history was noted. At the time of referral, she felt ill and had an elevated body temperature (38.3°C) under oral systemic clindamycin (day 4). The woman's heart rate was 105/min, her systolic blood pressure was 130 mmHg, and her respiration rate was normal. The surgical wound was clean with no signs of local infection. A systolic murmur was audible at Erb's point. C-reactive protein was 100 mg/L [normal range: 0.00–5.0 mg/L], procalcitonin was 0.83 ng/mL [normal range: 0.00–0.50 ng/mL], and the white blood cell count was elevated (14 G/L; [normal range: 4.4–11.3 G/L]). There were no Janeway lesions and no clinical signs of CNS embolization.

**Figure 1:**
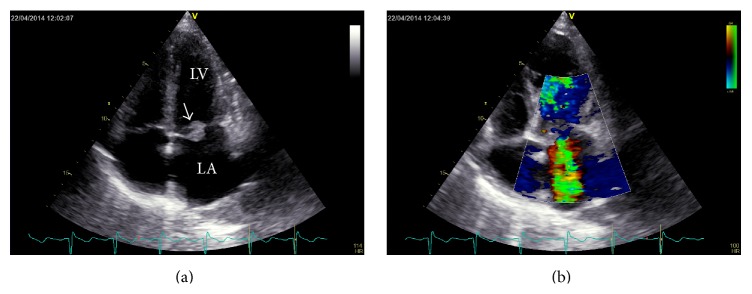
Transthoracic echocardiography (TTE), 4-chamber view of the heart. (a) The mitral valve is thickened and dysfunctional due to a floating vegetation (white arrow) on the anterior mitral leaflet; LA = left atrium; LV = left ventricle. (b) Color Doppler sonography shows severe mitral regurgitation.

Echocardiography showed a floating vegetation on the anterior mitral leaflet and severe mitral regurgitation (Figures 1(a)-1(b)). Pending the results of blood cultures obtained in the peripheral hospital and our institution, we started intravenous flucloxacillin (8 g per 24 hours IV in four divided doses) and penicillin (aqueous penicillin G 30 million units per 24 hours IV in three divided doses). An interdisciplinary panel of cardiologists, cardiac surgeons, infectiologists, and maternal-fetal medicine specialists was formed and the risks and benefits of early cardiac surgery versus waiting were explained to the patient in detail. The hemodynamically stable patient with increasing shortness-of-breath (NYHA II-III) was given time to consider the options. The mother of two and her partner agreed on early surgery, prioritizing treatment to protect her life over that of an extremely low birth weight infant (ELBW) with limited chances of survival. Sonographic assessment of the fetus revealed appropriate fetal growth and no signs of placental insufficiency or fetal malformations, so 12 mg betamethasone i.v. were administered on the same day to induce lung maturation.

**Figure 2:**
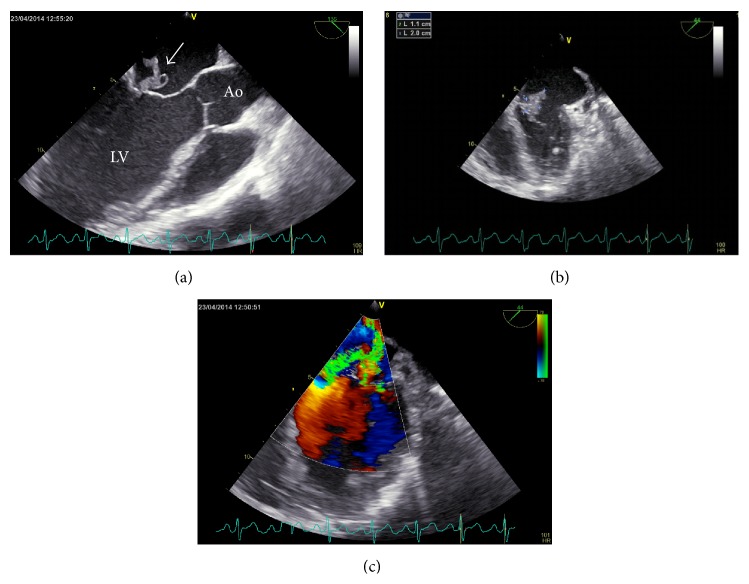
Transesophageal echocardiography (TEE): (a) 3-chamber view of the heart: floating vegetation (white arrow) on the anterior mitral leaflet; Ao = aorta; LV = left ventricle. (b) Another projection of the floating vegetation (11 mm × 20 mm) and the destroyed mitral valve. (c) Color Doppler sonography shows severe mitral regurgitation.

Transesophageal echocardiography (TEE) revealed a perforated anterior mitral leaflet and a vegetation of 20 mm × 11 mm (Figures 2(a)–2(c)). Both blood cultures tested positive for* Staphylococcus lugdunensis* (susceptible to oxacillin, resistant to penicillin and clindamycin). Paronychia of the toe was considered the most likely port of entry since* Staphylococcus lugdunensis* was also cultured from the wound swab obtained during surgery. Pencillin was stopped and flucloxacillin continued. Two interdisciplinary meetings also including anesthesiologists, neonatologists, plastic surgeons, and representative of the legal department were held to choose among the following three possible scenarios:

1. heart surgery without cesarean section;
2. cesarean section with subsequent heart surgery;
3. feticide and abortion with subsequent heart surgery.

While the postoperative risk of option 1 was considered too high, especially from the anesthesiologist's point of view, option 2 carried a high risk of massive hemorrhage. Option 3 was problematic due to the patient's worsening cardiovascular hemodynamics (NYHA III) subject to further destabilization following induction of labor. There was a consensus among all attendees and a consent of the patient and her partner on option 2, that is, cesarean section and subsequent heart surgery (artificial mitral valve replacement) followed by additional removal of both breast implants.

The surgery took place on April 24, 2014, within 48 hours of endocarditis diagnosis confirmation (20 hours after TEE and 45 hours after TTE, resp.). This was the sixth day after the initial wedge excision procedure and start of antibiotic treatment. The operation was performed in four consecutive phases. First (8:33 am–9:05 am), the female ELBW infant was delivered from breech presentation by caesarean section (weight: 505 g; APGAR: 1, 6, and 7 after 1, 5, and 10 minutes, resp.). Second (9:05 am–12:06 pm), the mitral valve was replaced by a mechanical valve during cardiopulmonary bypass under hypothermia (34°C) and cardioplegia. Third (12:15 pm–01:03 pm), the maternal abdomen was reopened to stop some minor bleeding and finally closed. Forth (1:52 pm–02:32 pm), both subpectoral breast implants including the capsular tissue (capsulectomy) were entirely removed.

The patient was transferred to the intensive care unit in a hemodynamically stable condition. TEE confirmed good positioning and function of the prosthesis.* Staphylococcus lugdunensis* was cultured from the resected mitral valve tissue. The patient survived the whole procedure without complications. However, after 72 h, the ELBW infant developed massive pulmonary and cerebral hemorrhage and succumbed on the 4th day.

The mother's postoperative course was uneventful. Two weeks after the heart surgery, she was clinically stable and transferred to a peripheral hospital. She was finally discharged four weeks later (after a total of 6 weeks of postoperative antibiotic treatment). Following the patient's request, a bilateral implant-based reaugmentation four months after the event was not recommended on the grounds of serious risk of infection. Ambulant follow-up visits 3, 5, 10, and 16 months after surgery showed that the patient is in good health, yet with persistent moderate perivalvular regurgitation of the artificial mitral valve.

## 3. Discussion

Maternal heart disease may complicate pregnancies and one rare but potentially fatal complication is infective endocarditis. Early diagnosis and appropriate antimicrobial treatment are critical to avoid neurological complications in infective endocarditis [2]. Published clinical guidelines addressing prophylaxis and management of infective endocarditis [6, 7, 9] do not extensively deal with concomitant pregnancy and relevant case reports are scarce.

Diagnosis and appropriate treatment of infective endocarditis depend on the identification of the causative microorganism [9]. Staphylococci and streptococci account for 80% of cases, with staphylococci being currently the most common pathogens [9]. At present,* Staphylococcus aureus* is both the leading cause and the most important prognostic factor for infective endocarditis [10]. To the best of our knowledge, ours is the first published report of infective endocarditis by* Staphylococcus lugdunensis* in a pregnant woman. Related case reports in the literature have described pregnant women presenting with endocarditis due to* Streptococcus viridans* [11] and* Streptococcus sanguinis* [12].

Cerebral complications are the most frequent and most severe extracardiac complications of infective endocarditis [9]. A multicenter observational cohort study on patients presenting with clear diagnoses of infective endocarditis has shown that the risk for embolism during infective endocarditis can be quantified on admission using multiple variables [13]. Vegetations that are large, mobile or are in the mitral position (all these criteria applied to our case) are associated with an increased risk of symptomatic embolism [9].

Surgical decision-making in infective endocarditis is largely consistent with established guidelines, although nearly 25% of patients with surgical indications do not undergo surgery [14]. While the timing and indications for surgical intervention to prevent systemic embolism in infective endocarditis remain controversial [15], there is no doubt that early valve surgery reduces the incidence of embolism in high-risk patients with endocarditis. In a randomized trial that compared clinical outcomes of early surgery and conventional treatment in patients with infective endocarditis, early surgery significantly reduced the composite end point of death from any cause and embolic events by effectively decreasing the risk of systemic embolism [8]. These data guided our decision in favor of early mitral valve replacement.

In the above-quoted trial, the authors hypothesized that the benefits of surgical treatment would be maximized by performing surgery within 48 hours after randomization, because the risk of embolism has been reported to be particularly high during the first week after diagnosis [16, 17]. Indeed, the rate of embolism in the early-surgery group was markedly reduced, as compared with conventional treatment [8]. These data urged us to perform surgery within 48 hours after confirming the diagnosis of* Staphylococcus lugdunensis* endocarditis. In order to stay within the 48-hour window, fetal lung maturation needed to be induced.

It is noteworthy, that the majority of patients investigated in the abovementioned trial had streptococcal endocarditis and that only 10.5% (8/76) of the cases were caused by* Staphylococcus aureus* [8]. The fact that staphylococcal infections (as in our patient with* Staphylococcus lugdunensis*) cause more cerebral complications and exhibit higher mortality further endorsed our decision for immediate surgical intervention. Anyway, the decision to pursue early valve surgery should be individualized for each patient, based on infection-specific characteristics rather than on solely the microbiology of the causative pathogen [18]. Besides, the clinical prognosis also depends on the initial condition of the infected valve prior to the infection [19].

Cardiac surgery during pregnancy carries significant maternal and fetal risk. Despite the high fetal mortality, urgent surgery should be performed during pregnancy in women who present with heart failure due to acute regurgitation [7]. The maternal and neonatal outcomes of cardiopulmonary bypass during pregnancy were recently investigated in twenty-one pregnant patients identified in the Mayo Clinic surgical database who had undergone cardiothoracic surgery between 1976 and 2009 [20]. Among them, six had mitral valve repair/replacement and seven patients underwent cesarean section immediately prior to sternotomy, delivering viable infants (median gestational age: 31 weeks) [20]. Today, cardiothoracic surgery can be performed relatively safely during pregnancy [20], although cardiopulmonary bypass immediately postpartum could carry the risk of severe uterine bleeding. With this in mind, we prepared twenty units of packed red blood cells and three platelet concentrates before our patient underwent surgery.

Our patient's breast implants were removed simultaneously to prevent reinfection. In women with breast implants, late infection usually results from secondary bacteremia or an invasive procedure at a location other than the breasts [21]. The patient's request for implant-based reaugmentation carried a significant but preventable risk of reinfection, and she was appropriately advised.

## 4. Conclusions

In conclusion, this is the first published report of infective endocarditis by* Staphylococcus lugdunensis* in a pregnant woman. Symptoms occurred in a critical stage of pregnancy and required swift interdisciplinary counsel and action on the part of representatives of seven specialties. Literature allowing for unambiguous therapeutic decisions in this constellation was scarce. An optimal outcome in a challenging case like this greatly depends on effective interdisciplinary communication, informed consent of the patient, and concerted action among the specialists involved.
